# High fat diet induced atherosclerosis is accompanied with low colonic bacterial diversity and altered abundances that correlates with plaque size, plasma A-FABP and cholesterol: a pilot study of high fat diet and its intervention with *Lactobacillus rhamnosus* GG (LGG) or telmisartan in ApoE^−/−^ mice

**DOI:** 10.1186/s12866-016-0883-4

**Published:** 2016-11-08

**Authors:** Yee Kwan Chan, Manreetpal Singh Brar, Pirkka V. Kirjavainen, Yan Chen, Jiao Peng, Daxu Li, Frederick Chi-Ching Leung, Hani El-Nezami

**Affiliations:** 1 5S12, Kadoorie Biological Sciences Building, School of Biological Sciences, The University of Hong Kong, Pokfulam, Hong Kong; 2 5N01, Kadoorie Biological Sciences Building, School of Biological Sciences, The University of Hong Kong, Pokfulam, Hong Kong; 3 Food and Research Health Centre, University of Eastern Finland, Joensuu, Finland; 4 L943, Laboratory Block, Department of Surgery, LKS Faculty of Medicine, The University of Hong Kong, 21 Sassoon Road, Pokfulam, Hong Kong; 5 Bioinformatics Center, Nanjing Agricultural University, Nanjing, China; 6 Institute of Public Health and Clinical Nutrition, University of Eastern Finland, Kuopio, Finland; 7 5S13, Kadoorie Biological Sciences Building, The University of Hong Kong, Pokfulam, Hong Kong

**Keywords:** Atherosclerosis, Probiotics, LGG, Telmisartan, Gut microbitoa

## Abstract

**Background:**

Atherosclerosis appears to have multifactorial causes – microbial component like lipopolysaccharides (LPS) and other pathogen associated molecular patterns may be plausible factors. The gut microbiota is an ample source of such stimulants, and its dependent metabolites and altered gut metagenome has been an established link to atherosclerosis. In this exploratory pilot study, we aimed to elucidate whether microbial intervention with probiotics *L. rhamnosus* GG (LGG) or pharmaceuticals telmisartan (TLM) could improve atherosclerosis in a gut microbiota associated manner.

**Methods:**

Atherosclerotic phenotype was established by 12 weeks feeding of high fat (HF) diet as opposed to normal chow diet (ND) in apolipoprotein E knockout (ApoE^−/−^) mice. LGG or TLM supplementation to HF diet was studied.

**Results:**

Both LGG and TLM significantly reduced atherosclerotic plaque size and improved various biomarkers including endotoxin to different extents. Colonial microbiota analysis revealed that TLM restored HF diet induced increase in Firmicutes/Bacteroidetes ratio and decrease in alpha diversity; and led to a more distinct microbial clustering closer to ND in PCoA plot. Eubacteria, Anaeroplasma, Roseburia, Oscillospira and Dehalobacteria appeared to be protective against atherosclerosis and showed significant negative correlation with atherosclerotic plaque size and plasma adipocyte – fatty acid binding protein (A-FABP) and cholesterol.

**Conclusion:**

LGG and TLM improved atherosclerosis with TLM having a more distinct alteration in the colonic gut microbiota. Altered bacteria genera and reduced alpha diversity had significant correlations to atherosclerotic plaque size, plasma A-FABP and cholesterol. Future studies on such bacterial functional influence in lipid metabolism will be warranted.

## Background

Atherosclerosis is the major cause of myocardial infarction and stroke, which accounts to the leading cause of death worldwide [1, 2]. Atherosclerosis is a chronic inflammatory disease of the arteries – inflammation is present and mediated by different chemokines/cytokines at all stages – from leukocytes recruitment by adhesion molecules in plaque formation to collagen cap digestions by metalloproteinases (MMPs) that contributes to the plaque instability.

Recently, there is more evidence that associates the gut microbiota to atherosclerosis. Endotoxin has become a popular candidate as the initiator of obesity and insulin resistance [3], not only can gut microbiota modulate endotoxemia [4], endotoxemia can also be resulted from a weakened gut barrier where endotoxin (LPS) and other bacterial products can easily leak into the circulation and trigger inflammatory response. Gastrointestinal (GI) track has been suggested to be the major site for pathogen associated molecular patterns (PAMPs) absorption and translocation [5]. Toll like receptors (TLRs) compose a family of recognition receptors for PAMPs, playing important roles in eliciting innate and adaptive immune responses [6]. TLR2, TLR4 and TLR5 can recognize and be activated by bacterial components found in the gut microbiota – peptidoglycan, LPS, and flagella respectively [7]. Inflammatory responses are augmented upon the activation of TLRs by NFκB and transcriptional activation of genes that encode pro-inflammatory cytokines and chemokines. With obesity and insulin resistance being major risk factors for atherosclerosis and cardiovascular diseases [8], it is not surprising to find endotoxin capable in accelerating and acts as a potent mediator for atherosclerosis [9–11]. Furthermore, presence and correlations of bacterial DNA in the atherosclerotic plaque to their abundance found in the GI tract had provided further support to potential causative links of the gut microbiota to atherosclerosis [12]. Investigations on modulating the gut microbiota in an attempt to reduce cardiovascular risks had increased significantly in the last decade – from realizing the potential of probiotics in improving lipid profile [13–16] and atherosclerotic plaque size [17, 18], and to finding the gut metagenome having a different functional capacity in symptomatic atherosclerotic patients [19], to recently identifying the gut microbiota dependent metabolites that significantly increase cardiovascular risks [20], have all contributed in assuring the metabolic role of gut microbes in atherosclerosis.

In this pilot study, we aimed to investigate whether probiotics and pharmaceutical interventions could improve atherosclerosis in a gut microbiota associated manner in a well-established atherosclerotic animal model, ApoE^−/−^ mice [21–23]. *L. rhamnosus* GG (LGG), one of the best clinically-documented probiotic strains [24], was first isolated more than 20 years ago by Goldin and Gorbach from a faecal sample from a healthy adult and showed to have high resistance against gastric acid and high persistence capacity in the human GI tract [25]. As it has proven benefits in various diseases including diarrhea [26, 27], colitis [28] and atopic disease [29, 30]; and its high adaptability in the GI tract, LGG is often regarded as a model probiotic strain [31]. To better compare the capability and the extent to how LGG may improve atherosclerosis, a positive control was introduced by the pharmaceutical intervention of telmisartan (TLM). TLM is a dual angiotensin II receptor blocker and partial perixosome proliferator-activated receptor Ɣ (PPARƔ) agonist. Clinically, TLM is used to treat hypertension, including patients with atherosclerosis [32], and can improve endothelial [33] and cardiovascular [34] functions. In vivo, it was proven to relieve atherosclerosis in the ApoE^−/−^ mice [35–37], and had recently been documented to reduce colonic inflammation in rats with inflammatory bowel disease [38]. We examined the effects of LGG and TLM supplementation in HF diet in terms of several atherosclerosis parameters and colonic gut microbiota, and identified some potentially important gut microbes in the pathogenesis of atherosclerosis.

## Methods

### Animals

Six-weeks-old female ApoE^−/−^ mice were fed *ad libitum* on a normal chow diet for 1 week before being continued at normal chow diet (ND) (D10001, Research Diet) or powdered 21gm% high fat diet (D12079B, Research Diet) without (HF) or with *Lactobacillus rhamnosus* GG (LGG) (ATCC 53103) (Vailo) (HF + LGG) or telmisartan (TLM) (Micardis®, Boehringer Ingelheim GmbH) at 5 mg/kg/day (HF + TLM) for 12 weeks. Ingredients of the ND and HF diets can be found in Tables 1 and 2. For this pilot study, the number of mice used in ND, HF, HF + LGG and HF + TLM groups were 5, 4, 3 and 5 respectively. The dose of LGG at 1×10^8^ CFU/day was converted from the recommended human dose using the Body Surface Area normalization method [39]. Lyophilized LGG powder was mixed in the HF diet; TLM was mixed into the drinking water – oral gavage was avoided to prevent stress induced immunomodulation. Fecal recovery of LGG has been tested to be at approximately 1×10^6^CFU/g by fecal dilution and plating on LB plates (data not shown). Food and water consumption was monitored twice a week to adjust LGG or TLM dosages. All animals were kept in the Animal Laboratory of the Department of Surgery at 23–24 °C and relative humidity at 60–70 % on 12/12 h day/night cycle. All study protocols were approved by the Committee on the Use of Live Animals in Teaching and Research (CULATR) of the University of Hong Kong and the Department of Health of the HKSAR Government.

**Table 1:** Protein, carbohydrate and fat content of ND and HF diet used in this pilot study

	ND		HF	
	gm %	kcal %	gm %	kcal %
Protein	20.3	20.8	20	17
Carbohydrates	66	67.7	50	43
Fat	5	11.5	21	41
Total		100		100
kcal/gm	3.9		4.7

**Table 2:** Ingredients of ND and HF diet used in this pilot study

	ND		HF	
Ingredient	gm	kcal	gm	kcal
Caesin 30 Mesh	200	800	0	0
Caesin, 80 Mesh	0	0	195	780
DL-Methionine	3	12	3	12
Corn starch	150	600	50	200
Maltodextrin 10	0	0	100	400
Sucrose	500	2000	341	1364
Cellulose, BW200	50	0	50	0
Milk fat, anhydrous	0	0	200	1800
Corn oil	50	450	10	90
Mineral Mix S10001	35	0	35	0
Calcium carbonate	0	0	4	0
Vitamin Mix V10001	10	40	10	40
Choline Bitartrate	2	0	2	0
Cholesterol, USP	0	0	1.5	0
Ethoxyquin	0	0	0.04	0
Total	1000	3902	1001.54	4686

### Quantification of plasma biomarkers

Mice were fasted overnight before sacrifice. Blood was collected in tubes coated with EDTA followed by centrifugation at 5,000 rpm for 10 min. Plasma was collected, aliquoted and stored at −80 °C until use. The plasma concentration of the following biomarkers were quantified using Milliplex™ MAP kits and Luminex 200 Analyzer (Merck Millipore) following the manufacturer’s protocol: ghrelin with Milliplex®MAP Mouse Gut hormone Panel; sE-selectin, MMP-9, sICAM-1 and sVCAM-1 with Milliplex®MAP Mouse Cardiovascular Disease Panel I kit; IL-33 with Milliplex®MAP Mouse Cytokine/Chemokine Panel III Immunoassay respectively; with data acquisition and basic data analysis using xPONENT 3.1 and advanced data analysis using Milliplex Analyst 3.5. Plasma A-FABP and cholesterol was quantified using Mouse Adipocyte FABP ELISA Kit (BioVendor Research and Diagnostic Products Brno) and Cholesterol Assay Kit (Cayman Chemical Company) respectively following manufacturer’s protocol. Plasma endotoxin was quantified with Lonza Limulus amebocyte lysate (LAL) QCL-1000 (Lonza) following manufacturer’s protocol with heat inactivation at 75 °C for 10 min and addition of β-glucan blocker (Lonza) (1:1) to inhibit false positive readings generated by contamination from β-1,3-glucans.

### Assessment of atherosclerotic plaque size

#### Aortic sinus

Hearts were harvested, fixed in 4 % PFA overnight, embedded in Tissue-Tek® OCT compound (Sakura Finetek) and stored at −20 °C before cryosectioned serially at 10 μm intervals from the aortic sinus and mounted on slides (Superfrost plus). Oil Red O Solution was freshly prepared as previously described [40] and used within 2 h of preparation. Slides were stained with Leica ST5020 Multistainer (Leica Microsystems Inc) for better consistency. Briefly, slides were immersed in tap water for 2 min, 60 % isopropanol for 30s, Oil Red O Solution for 18 min, 60 % isopropanol for 30s, tap water for 2 min, hematoxylin for 2 min, tap water for 3 min, 2 % acetic acid for 3 s, tap water for 20 s, Bluing solution for 1 min and tap water for 1 min before air drying. Images for viewed and captured with Nikon 80i Microscope and lesion quantified using SPOT™ Software 4.7.

#### Aortic tree

Fat tissue and adventitious blood vessels were removed from the inferior vena cava carefully with micro-scissors and micro-dissection forceps under dissecting microscope. The inferior vena cava was harvested and stored in PBS at 4 °C until use. Sudan IV solution was prepared by preparing 0.5 % Sudan IV in 35 % ethanol and 50 % acetone followed by filtration. The artery was placed flat on a Petri dish, first rinsed with 70 % ethanol, stained with Sudan IV solution for 5 min and de-stained in 80 % ethanol for 6 min before being viewed under a Zoom Stereo Microscope (Olympus SZX7®) and images captured with an Olympus DP71 Microscope Digital Camera. Areas stained red were considered atherosclerotic lesions and were quantified using Image J software.

#### Colonial gut microbiota evaluation

DNA was extracted from colon using QIAamp® DNA Stool Mini Kit (Qiagen) followed by amplification of the V4-6 region of the 16S rRNA gene at position 563 to 1064 with a product size of 573 bp with Expand High Fidelity^PLUS^ PCR System (Roche). Each 25 μl of PCR mixture contained: 1× Expand High Fidelity^PLUS^ Reaction Buffer with 1.5 mM MgCl_2_; 200 μM PCR Grade Nucleotide Mix; 0.4 μM of the forward and reverse primers, 1.25U of Expand HiFi^PLUS^ Enzyme Blend and 5–500 ng of template genomic DNA isolated from the mouse colon. The PCR protocol involved denaturation at 95 °C for 2 min; 35 cycles of denaturation at 95 °C for 20 s, annealing temperature at 53 °C for 30 s and extension at 72 °C for 40 s; and a final extension at 72 °C for 10 min. Target PCR products were verified on 1 % agarose gel with TAE buffer. A band of 573 bp was gel-purified with QIAquick® Gel Extraction Kit (Qiagen) and the final concentration of the amplicon was quantified using Quant-iT™PicoGreen® dsDNA assay (Invitrogen). Library preparation, emPCR amplification and picotitre plate pyrosequencing using titanium chemistry was carried out in accordance with Roche/454 Life Sciences protocols on the 454 GS Junior (454 Life Sciences-a Roche Company). A total of 397,743 sequence reads were generated, with an average of 23,397 reads per sample. All sequence reads derived from the 454 GS Junior pyrosequencer were analyzed using Quantitative Insights Into Microbial Ecology (QIIME) [41]. Prior to sequence analysis, all raw reads were denoised to reduce sequencing errors and avoid artificial inflation of OTU diversity and processed for quality trimming. Quality trimming parameters included removal of forward and reverse primer sequences, retaining a minimum sequence length of 150 bp; maximum sequence length of 573 bp; minimum quality score of 25 in a 50 bp sliding window. Quality trimmed reads were demultiplexed based on pre-designed barcodes followed by determination of operational taxonomic units (OTUs) by clustering sequences with a similarity threshold of 97 % using uclust. Representative sequences were picked and aligned using MUSCLE (Multiple Sequence by Log-Expectation) against the Greengenes Core Set data followed by taxonomy assignment to each sequence using the Blast algorithm after screening and eliminating chimeric reads. Subsequent analysis included relative and absolute abundance of bacteria, alpha and beta diversity and principal coordinates (PCoA) plots based on unweighted UniFrac using inherent scripts within the QIIME package. All 454 sequence data associated with this study has been deposited in the NCBI SRA repository under the study accession no. SRP026050.

#### Statistical analysis

Data are expressed as mean ± SEM. Data normality was analyzed using Shapiro-Wilk test [42]. Kruskal-Wallis Test with Dunns post hoc tests were used to analyze nonparametric data; One Way ANOVA with Bonferroni post hoc tests were used to analyze parametric data. Statistical tests were done using GraphPad Prism 5. Correlation studies were done using R [43]. *P* < 0.05 was considered statistically significant.

## Results

### LGG and TLM significantly reduced high fat diet induced atherosclerosis plaque size at aortic sinus and aortic tree

**Fig. 1:**
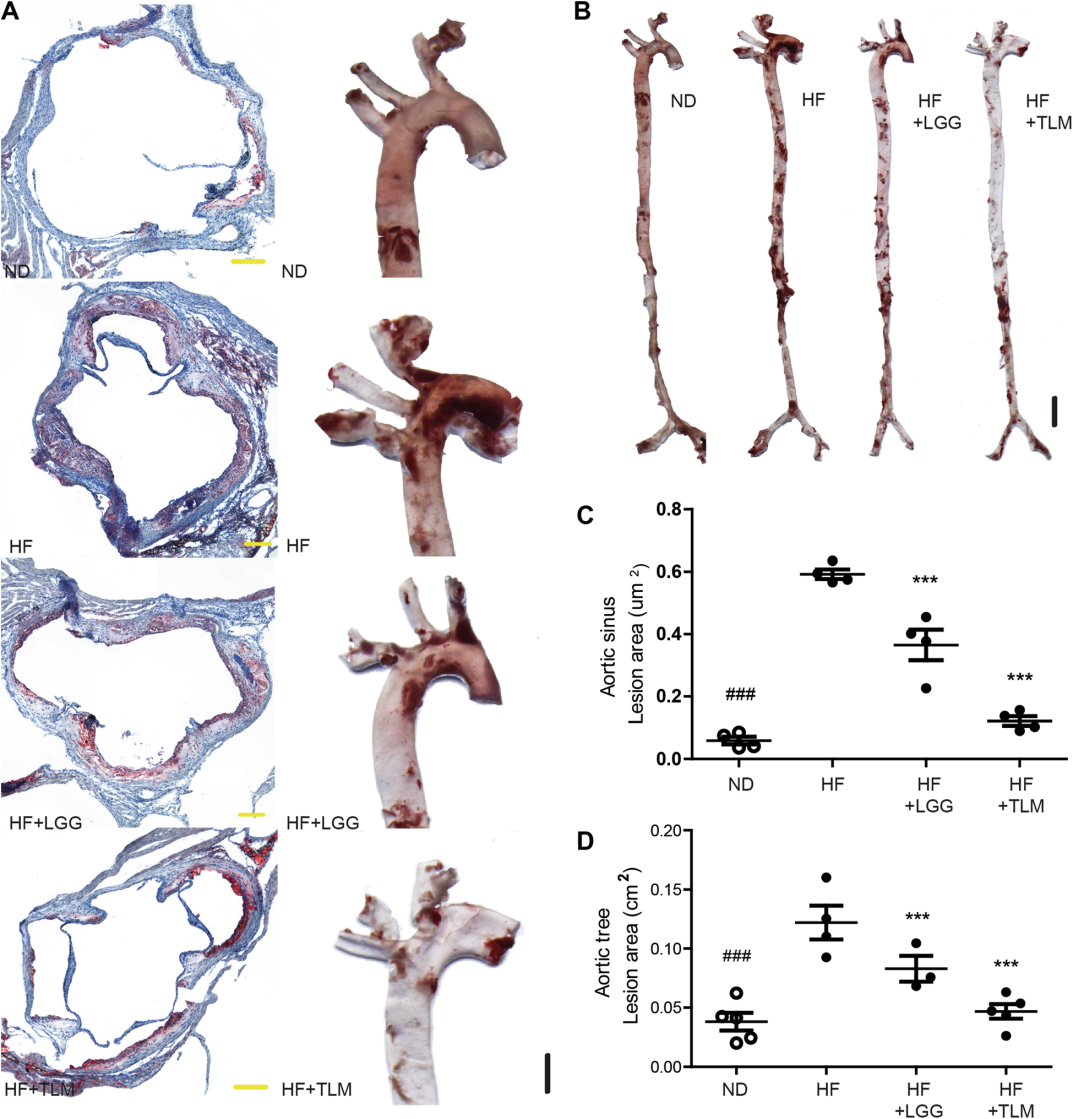
Atherosclerotic plaque characterization. Lipid was stained red with Oil Red O at the aortic sinus (**a**, LHS), and Sudan IV in the aortic tree (**b**) (Ascending aorta, arch of aorta, part of the descending aorta, brachiocephalic artery, left common carotid artery and left subclavian artery was magnified at the RHS of **a**); the size of the lesion area at the aortic sinus (**c**) and the entire aortic tree (**d**) was quantified using ImageJ. Significant difference of ND from the HF group was denoted by ^###^ *P* < 0.001; significant difference of the treatment groups from HF group was donated by ***P < 0.001. ApoE^−/−^ mice were fed on either normal chow diet (ND) or high fat (HF) diet with or without LGG (1×10^8^CFU/day) or TLM (5 mg/kg/day)

The lesion at the aortic root (Fig. 1a LHS) and aortic tree (Fig. 1b and magnified at Fig. 1a RHS) was stained red with Oil Red O and Sudan IV respectively. 12 weeks of HF diet significantly increased lesion area in ApoE^−/−^ mice at the aortic sinus by 8 fold, from a mean for ND at 0.06 mm^2^ to HF at 0.59 mm^2^. LGG and TLM supplementation reduced the lesion size by 37 % (0.37 mm^2^) and 80 % (0.12 mm^2^) respectively (Fig. 1c). Comparable results were seen at the aortic tree – the lesion area in HF was 2.21 fold higher than ND (0.12 cm^2^ vs 0.04 cm^2^, respectively), while LGG and TLM supplementation led to a 33 % (0.08 cm^2^) and 58 % (0.05 cm^2^) reduction respectively (Fig. 1d). TLM was effective in restricting the HF diet induced atherosclerotic plaques at the aortic sinus and the aortic tree to a level close to that is seen in the ND group.

### HF diet led to cholesterol crystals formation in plaque and worsened various atherosclerosis related biomarker levels which were improved by LGG and TLM to different extents

**Fig. 2:**
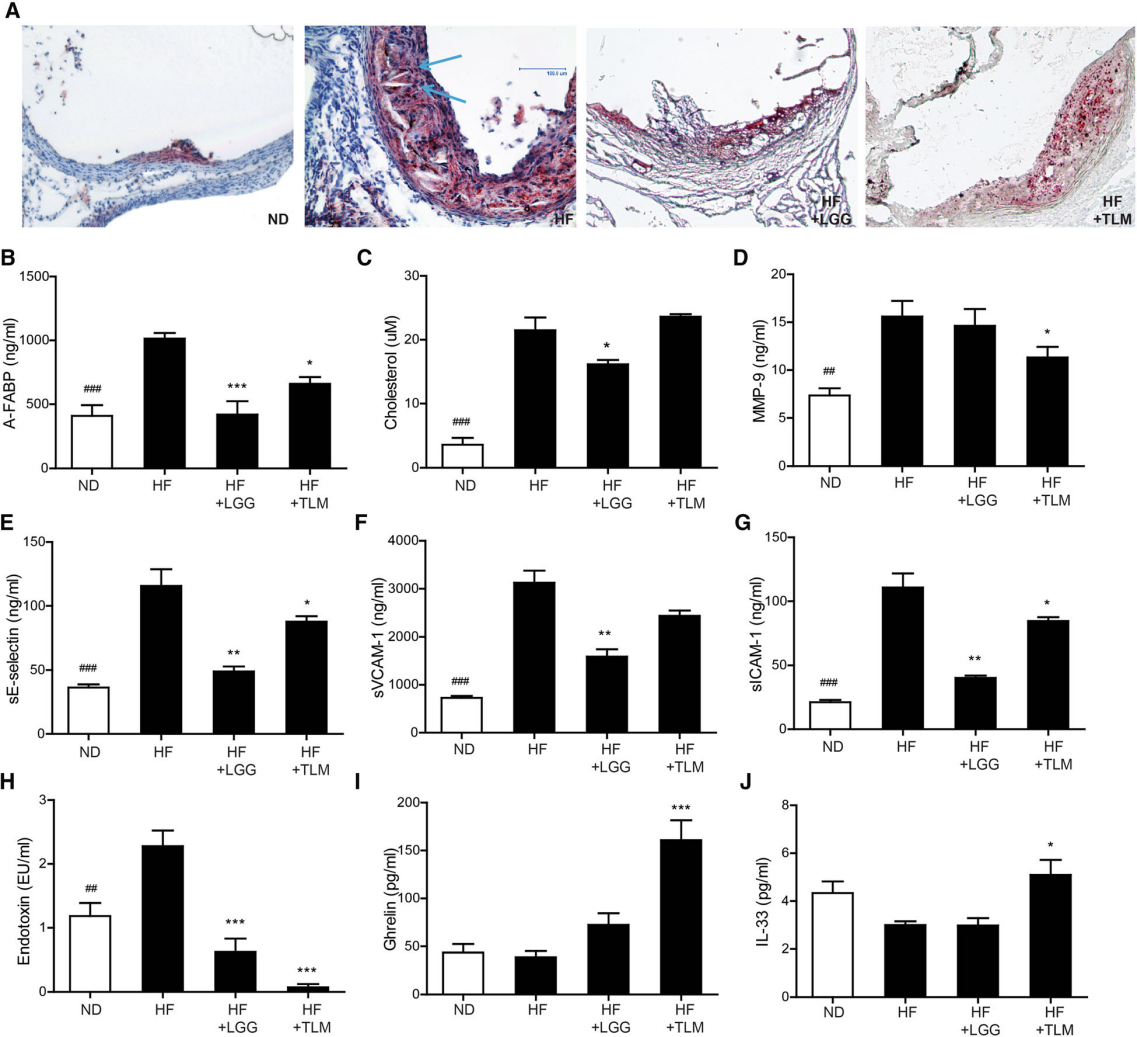
Cholesterol crystals in atherosclerotic plaque and plasma concentrations of atherosclerosis related biomarkers. Atherosclerotic plaque stained with Oil Red O at the aortic sinus, with blue arrows indicating the cholesterol crystals (**a**). Plasma concentration of A-FABP (**b**); cholesterol (**c**); MMP-9 (**d**); sE-selectin (**e**); sVCAM-1 (**f**); sICAM-1 (**g**); endotoxin (**h**); ghrelin (**i**) and IL-33 (**j**). Significant difference of ND from the HF group was denoted by ^#^ *P*P < 0.05, ^##^ *P* < 0.01, ^###^ *P* < 0.001; significant difference of the treatment groups from HF group was donated by *P < 0.05, **P < 0.01, ***P < 0.001. ApoE^−/−^ mice were fed on either normal chow diet (ND) or high fat (HF) diet with or without LGG (1×10^8^CFU/day) or TLM (5 mg/kg/day)

**Fig. 3:**
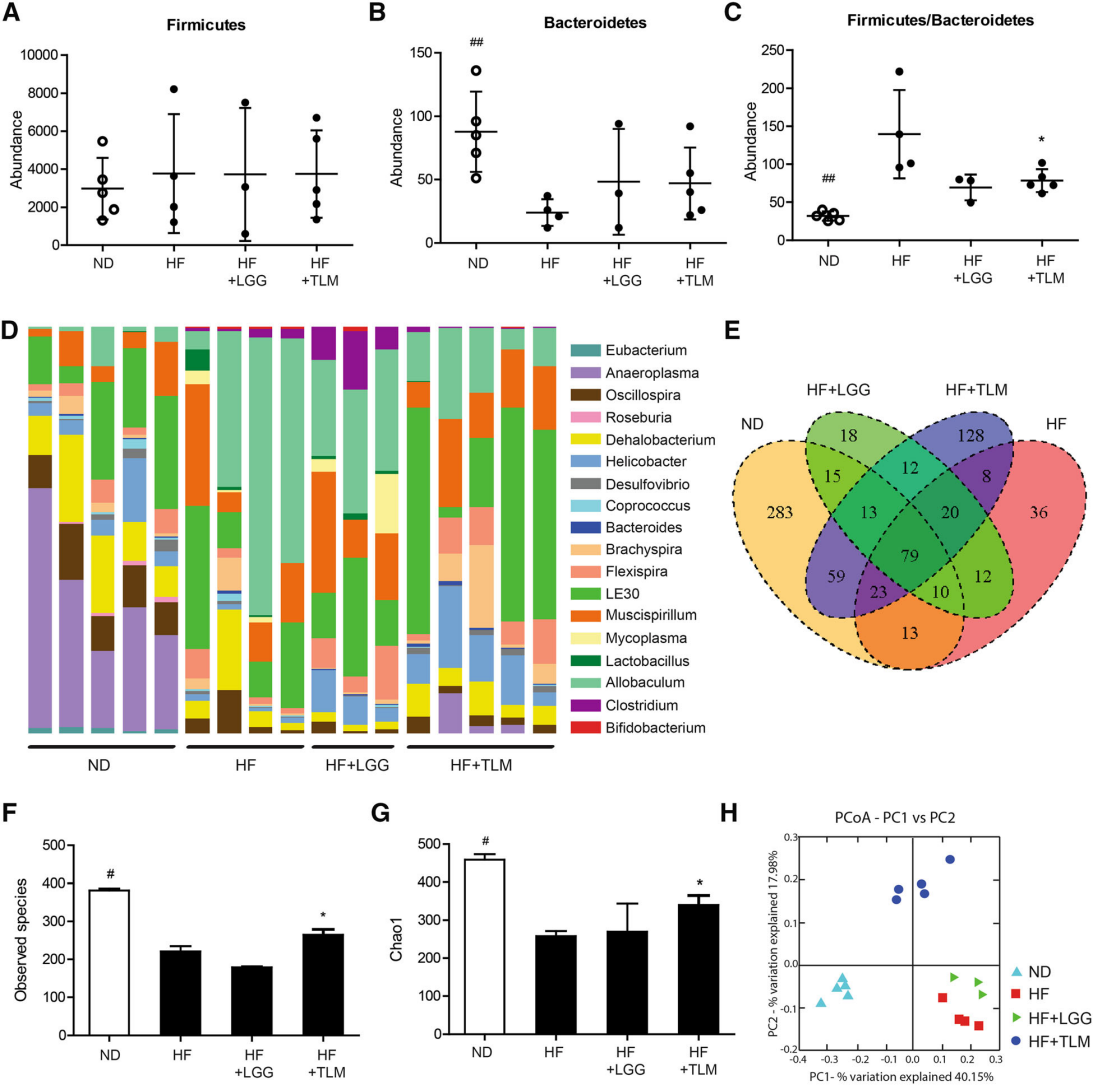
Colonic gut microbiota alterations. Absolute abundances of (**a**) Firmicutes, (**b**) Bacteroidetes and the ratio of Firmicutes over Bacteroidetes (**c**); relative abundances of 18 bacteria at genus level with raw counts of at least 100 (**d**); Venn diagram summarizing number of OTUs shared between different groups (**e**); alpha diversity indexes including number of observed species (**f**) and Chao1 index (**g**); beta diversity indicated by PCoA plot of unweighted UniFrac distance showing sample clustering by treatment groups (**h**). Significant difference of ND from the HF group was denoted by ^#^ *P* < 0.05, ^##^ *P* < 0.01; significant difference of the treatment groups from HF group was donated by *P < 0.05. ApoE^−/−^ mice were fed on either normal chow diet (ND) or high fat (HF) diet with or without LGG (1x10^8^CFU/day) or TLM (5 mg/kg/day)

Atherosclerosis is associated with worsened lipid and adipokine profile, as well as various inflammation related biomarkers. HF diet led to formation of cholesterol crystals in the atherosclerotic plaque that were not observed in the other groups (Fig. 2a). HF diet significantly elevated plasma levels of A-FABP, cholesterol, MMP-9, sE-selectin, sICAM-1, sVCAM-1 and endotoxin. Supplementing LGG in HF diet significantly decreased such elevation except MMP-9; while TLM reduced the plasma levels of A-FABP, MMP-9, sE-selectin, sVCAM-1 and endotoxin (Fig. 2, b, c, d, e, f, g & h). On the other hand, while HF diet did not induce any significant change in plasma level of ghrelin nor IL-33, they were significantly increased by TLM supplementation (Fig. 3h, i).

### HF diet distorted colonial microbial profile and reduced bacterial diversity, which were counteracted by TLM but not LGG

In assessing statistically significant alterations in the bacterial community, only those taxa for which raw counts reached at least 100 were considered. At the phylum level, while no treatment group led to significant change in the abundance of Firmicutes (Fig. 3a), HF diet in general had led to significant decrease in Bacteroidetes (Fig. 3b). The ratio Firmicutes/Bacteroidetes (F/B), which usually increases during obese states and dysbiosis [44, 45], was markedly elevated by HF diet. Supplementing LGG and TLM in HF diet trended and significantly lowered F/B respectively (Fig. 3c). At the genera level, HF diet had led to significantly lower colonial abundances of Eubacterium, Anaeroplasma, Oscillospira, Roseburia and Dehalobacterium and higher abundances of Allobaculum, Clostridum, Lactobacillus and Bifidobacteria when compared to ND. The HF + LGG group had significantly higher Lactobacillus and Clostridum abundances compared to the ND and HF + TLM group; whereas the HF + TLM group had significantly higher Helicobacter abundance than HF alone (Fig. 3d).

The degree of OTUs shared between individual mice and the four groups was summarized in the Venn diagram (Fig. 3e). Greatest numbers of OTUs were shared between mice in the same group. With respect to the ND-HF comparison, 125 OTUs were common to both groups while a large number of OTUs were still unique to each group (370 for ND; 76 for HF). The HF + TLM group (174) had a greater overlap with the ND group OTUs compared to the HF + LGG group (117), revealing a more potent effect in maintaining normal bacterial composition in the colon. The core microbiome comprised 79 OTUs that were present regardless of manipulations to the diet.

Overall bacterial species diversity within the four groups was examined using alpha diversity indexes including observed number of species (Fig. 3f) and the chao1 index (Fig. 3g). The HF diet significantly reduced the number of observed species (221 ± 27) compared to the ND (381 ± 11). While the LGG supplementation did not change the number of observed species (179 ± 4) compared to HF diet alone, significant increase was seen with TLM supplementation (265 ± 31). The number of observed species in both the HF + LGG and HF + TLM groups remained significantly lower than the ND group (Fig. 3f). The impact of the HF diet was similarly indicated (reduction in diversity) through the Chao1 index (Fig. 3g). The significant higher number of observed species and Chao1 index suggests that species richness in the ND group was populated by the presence of many more rare species. The unweighted UniFrac PCoA plot revealed clear microbial diversity demarcation resulting from a HF diet compared to the ND group. Mice of HF + LGG or HF + TLM groups aggregated into distinct clusters as well based on their bacterial communities (Fig. 3h).

### Lowered bacterial diversity and alterations in several colonic bacterial genera highly associated with changes in several atherosclerosis parameters

**Fig. 4:**
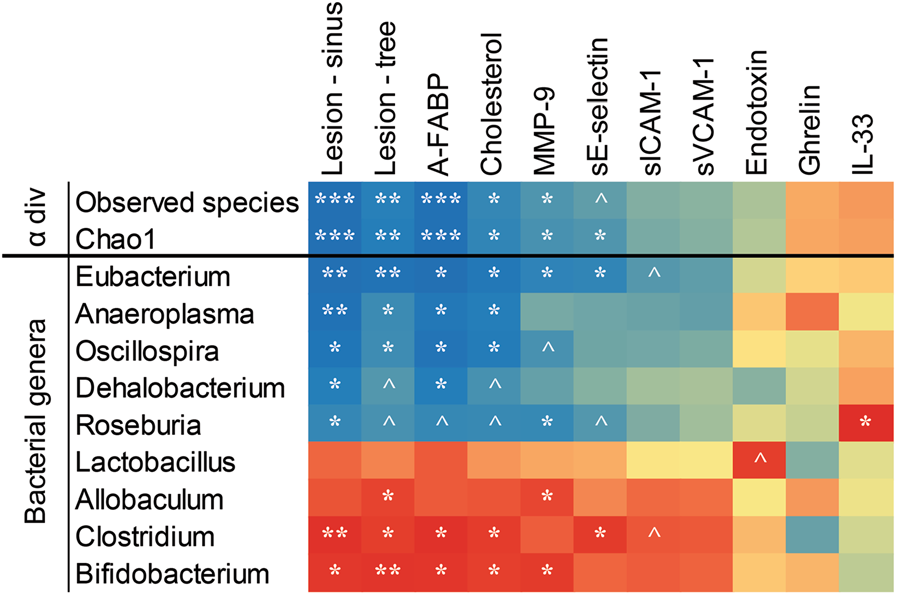
Heat map describing the correlation of the alpha diversity and abundances of different colonic bacterial genera and atherosclerosis parameters. Bacteria genera shown were significantly altered (*P* < 0.05) by HF diet feeding in ApoE^−/−^ mice. The colors range from blue (negative correlation; −1) to red (positive correlation; +1). Significant correlations were noted by *P < 0.05, **P < 0.01 and ***P < 0.001; strong tendency of correlation was noted by ^*P* < 0.10

As ND and HF had the most drastic differences detected in atherosclerosis lesion size (Fig. 1), related biomarkers (Fig. 2) and gut microbial abundances (Fig. 3), correlations between the atherosclerotic parameters and bacterial taxa were investigated between the groups (Fig. 4). Only bacterial genera with an accumulative count of at least 100 across all samples were studied. Of these, genera that showed statistically significant abundance difference between ND and HF group were selected for the correlation analysis. The HF diet induced significantly lowered number of observed species and chao1 index; as well as the colonic abundances of Eubacterium, Anaeroplasma, Oscillospira, Dehalobacterium and Roseburia (*p* < 0.05) showed significant negative correlations to the increase in lesion size at the aortic sinus (*p* < 0.05). Moreover, the reduction of the number of observed species, chao1 index and abundances of Eubacterium, Anaeroplasma and Oscillospira showed significant negative correlations with lesion area of the entire aortic tree, plasma A-FABP and cholesterol. Reduction in Eubacterium also showed significant negative correlation with increase in MMP-9 and sE-selectin; similar observations were also seen between Dehaobacterium with A-FABP and Roseburia with MMP-9. Notably decrease in Roseburia showed positive correlations to the decrease in plasma IL-33. (Fig. 4). In contrast, HF diet induced significant increase in abundances of Lactobacillus, Allobaculum, Clostridium and Bifidobacterium (*p* < 0.05), of which Clostridium and Bifidobacterium showed significant positive correlations with atherosclerotic lesion size at the sinus, aortic tree, plasma A-FABP, cholesterol, MMP-9 or sE-selectin (Fig. 4).

Likewise, when correlations were analyzed between HF & HF + LGG groups or HF & HF + TLM groups, significant or strong tendency of similar correlations as noted between ND & HF groups were observed, especially between Eubacterium, Dehalobacterium, Roseburia and Clostridium and lesion size, A-FABP and cholesterol (Data not shown). Such correlations were not as strong probably due to as compared to HF & ND, supplementing LGG or TLM to HF induced a relatively smaller changes in atherosclerotic parameters and changes in bacterial genera abundances.

## Discussion

From the earlier studies that showed bacterial abundances in atherosclerotic plaques correlating with those in the gastrointestinal tract [12]; patients with symptomatic atherosclerosis having an altered gut microbial abundances and metagenome that was enriched in genes encoding peptidoglycan synthesis but depleted in those for phytoene dehydrogenase [19]; to recently identifying gut microbiota dependent metabolites including trimethylamine-N-oxide (TMAO) and Ɣbutyrovbetaine (ƔBB) from dietary phosphatidylcholine and L-carnitine, that serve as strong predictors of coronary artery disease [20, 46], have all suggested the gut microbiota as a very potential therapeutic target for reducing atherosclerosis associated cardiovascular disease risks.

In this pilot study, we have first induced a strong atherosclerotic phenotype with HF diet in ApoE^−/−^ mice – including significantly increase in atherosclerotic plaque size and undesirable levels of atherogenic biomarkers; then we compared the effects induced by microbial (LGG) and pharmaceutical (TLM) intervention in terms of gut microbial changes and different atherosclerosis associated parameters. One limitation of the current study is the relatively small sample size. As a pilot study, a smaller animal numbers is typically used to generate data as the foundation for future experiments. For example, exciting insights have been generated from some pilot studies with limited number of animals for further studies [47, 48]. With our previous experience in the lesion size expected in normal chow or high fat diet in the ApoE^−/−^ mice, the current number of mice used were justified in generating meaningful statics [49]. In essence, we have found the following from the pharmaceutical or probiotic intervention of gut microbiota in relevance to the development of atherosclerosis:

Firstly, we found that both LGG and TLM could significantly reduce HF diet induced atherosclerotic plaque size, where TLM had a stronger plaque reducing power (Fig. 1). While TLM is used as a control to reduce atherosclerotic plaque size in ApoE^−/−^ mice [35–37], certain probiotics including *L. acidophilus* ATCC 4358 [18] and *Enterococcus faecium* CRL183 & *L. helveticus* 416 [17] had been previously shown to reduce atherosclerotic plaque size in ApoE^−/−^ mice and rabbits on hypercholesterolemic diet respectively. Such reduction might be modulated through lowering oxidative stress and inflammation [18].

Secondly, we showed that atherosclerotic improvement led by LGG and TLM were indicative by several associated biomarkers. LGG could reduce the HF diet induced lipid related parameters including cholesterol and A-FABP, an adipokine that is an important player in lipid metabolism and a pathophysiological mediator of atherosclerosis [50]. Cholesterol reduction has been implied for lesser cardiovascular events owing to the resultant anti-inflammatory effects, improved plaque stability and reduced risk of thrombotic complications [51]. While some probiotics had shown hypocholesterolemic effects clinically [52, 53], the effects are likely to be strain specific as others have failed to show the same effect [54, 55]. Supplementation of LGG or TLM to HF diet also resulted in reduction of cholesterol crystals in the atherosclerotic plaque. As cholesterol crystals have been suggested to be capable of physically perforating cell membranes and the plaque cap, subsequently triggering cell apoptosis and plaque rupture respectively [56, 57], LGG and TLM could have further conferred plaque stability. LGG also reduced soluble E-selectin, ICAM-1 and VCAM-1, contributing to a less atherogenic environment. Furthermore, LGG reduced endotoxemia, a promising candidate that initiates obesity and insulin resistance [3]. Indeed, probiotics had been suggested in quite a few studies to be beneficial to cardiovascular system by improving lipid profiles [13, 16, 58] and endotoxemia [58]. One potential mechanism to how LGG improved lipid profile was its ability to produce exopolysaccharides [59]. Exopolysaccharides can serve as a protective shield for LGG to fight against the host innate immunity [60] and at the same time modify the enteric gut microbiota and reduce liver and serum cholesterol and triglyceride concentrations [59]. Certain *L. rhamnosus* strains can also produce specific delipidating molecules, such as conjugated linoleic acid [61, 62] that interfere with host metabolism by modulating energy expenditure, fatty acid oxidation, lipolysis and lipogenesis [61]. While LGG induced a relatively less alteration in the gut microbial abundances in this study, it should be noted that the lipid modulating effects exerted by Lactobacillus can be independent to its intestinal colonization [63].

TLM resulted in similar significant or trended reduction in the tested plasma biomarkers, especially in MMP-9 and endotoxin. The inhibitory effects of TLM on A-FABP [64, 65] and monocytic cell adhesions [66] may have been mediated by PPARƔ activation; and on MMP-9 [67–72] by Elk-1 phosphorylation inhibition [67]. While TLM was previously shown to lower cholesterol [73, 74], possibly through inhibiting intestinal cholesterol absorption [74], such reduction was not seen in the current study. Interestingly, TLM was able to increase plasma ghrelin and IL-33 concentrations, suggesting TLM may contribute to an anti-atherogenic environment by mediating Th1 to Th2 responses [75, 76].

Thirdly, we demonstrated that HF diet induced atherosclerosis was associated with a much lower colonic microbial diversity. The reduced microbial diversity may be from the selected growth of bacteria that better utilized fatty acids as their energy source, leading to their dominances and out-competing the growth of others. Resistance to pathogenic bacteria colonization may be reduced resulting in worsened gut barrier functions. Although LGG did not increase gut microbial diversity and a less distinct clustering in the PCoA plot that was further away from ND in this study, *L. plantarum* had been shown to increase bacterial diversity in the colon in men with incipient atherosclerosis [77]. The restoration of bacterial diversity by TLM suggests that supplementing TLM was able to blunt the drop in the microbial assemblage, its potential ability to sustain a more diverse bacterial community despite the same HF content.

Furthermore, we have identified five bacteria genera that are most likely protective against atherosclerosis – Eubacerium, Anaeroplasma, Oscillospira, Roseburia and Dehalobacterium. These microbes correlated strongly with atherosclerotic lesion area at the aortic sinus and aortic tree, plasma cholesterol, A-FABP and MMP-9. Recently, Eubacterium and Roseburia were found to be enriched in the gut microbiota in healthy controls against those with symptomatic atherosclerosis [19]. Eubacteria and Roseburia, in our pilot study, were at much higher abundance in the ND and HF + LGG groups compared to the HF group. As Eubacteria and Roseburia are butyrate producing bacteria [78, 79], they may inhibit proliferation of vascular smooth muscle cells [80] conferring athero-protective effects. Oscillospira is often found strongly increased in hosts fed on fresh green fields [81], and Anaeroplasma is correlated with better crude fiber digestibility [82]. Higher abundances of Oscillospira and Anaeroplasma may have conferred protective effects based on their capacity on fiber metabolism. For example, *O. guillermondii*, together with a more abundant network of primary fiber degraders were found associated with lower BMI [83].

On the other hand, HF diet had significantly enhanced the colonic population of Lactobacillus, Allobaculum, Bifidobacterium and Clostridium, among which the increase in Bifidobacterium and Clostridium were positively correlated to atherosclerotic plaque size, A-FABP and cholesterol. The main elevated Bifidobacteria species in the HF group was *B. pseudologum,* which is the most predominant Bifidobacterium species in the infant gut [84] but insufficiently understood. Interestingly, Bifidobacterium were also found to have a significant positive correlation with TMAO, the gut microbiota dependent metabolite that predicts CVD risks [85]. With the complete genome of *B. pseudologum* completed [86], the functional capacity of the Bifidobacterium genera should be further explored for its relationship with atherosclerosis. *Clostridium cocleatum*, which was significantly elevated in the HF group, can degrade mucin [87] thus could impair the gut barrier and thereby augment systemic inflammation. As for Allobaculum, which correlated positively with atherosclerotic plaque size and MMP-9 in our pilot study, and TMAO in others [85], was also associated with low fat [88] and prebiotics [89] feeding. Lactobacillus, while being elevated in the HF diet, did not have any significant correlation with the atherogenic parameters tested in this study. So far, certain Lactobacillus species had been associated with obesity and weight gains while others with weight loss, for example, *L. reuteri* enrichment and *L. casei/paracasei* and *L. plantanum* depletion in an obese gut metagenome [90]. The complete genomes of seven different weight gain or weight protection associated Lactobacillus were recently sequenced and genes involved in carbohydrates and lipid metabolism and bacteriocin were identified to have a prominent role in nutrients harvesting and defense against oxidative stress [91], thus contributing to their associations to pro/anti-obesity.

## Conclusion

In this pilot study, we showed that microbial intervention with LGG or pharmaceutical intervention with Telmisartan were effective in reducing atherosclerotic plaque size and improveing plasma levels of various atherosclerosis associated biomarkers to different extents. TLM was more effective than LGG in altering the gut microbiota – in terms of colonic bacterial diversity and abundances closer to that observed when fed a ND diet. A lowered colonic bacterial diversity, abundances of Eubacterium, Anaeroplasma, Roseburia, Oscillospira, Dehalobacterium and increased Clostridium and Bifidobacteria was associated with an adverse atherogenic profile, especially atherosclerotic plaque size, plasma levels of A-FABP and cholesterol. As A-FABP, a suggested nexus where nutritional and inflammatory pathways meet [92], and cholesterol are crucial players in lipid metabolism, future studies focusing on the functional capacities in lipid metabolism of the above-mentioned bacteria will be valuable in understanding their impact on atherogenic development and to understand whether they are an active conspirator to pathology or a silent reflection of disease progression/outcome.

## Abbreviations

A-FABP: Adipocyte fatty acid binding protein
ApoE−/−: Apolipoprotein E knockout
ƔBB: Ɣ-butyrobetaine
GI: Gastrointestinal
HF: High fat
LGG: *Lactobacillus rhamnosus* GG
LPS: Lipopolysaccharides
MMPs: Metalloproteinases
ND: Normal diet
OTU: Operational taxonomic units
PAMPs: Pathogen associated molecular patterns
PCoA: Principal coordinates
PPARƔ: Perixosome proliferator-activated receptor Ɣ
QIIME: Quantitative insights into microbial ecology
sE-selectin: Soluble E-selectin
sICAM-1: Soluble intercellular adhesion molecule – 1
sVCAM-1: Soluble vascular cell adhesion molecule – 1
TLM: Telmisartan
TMAO: Trimethylamine-N-oxide
